# Polymer-Based Conductive Nanocomposites for the Development of Bioanodes Using Membrane-Bound Enzyme Systems of Bacteria *Gluconobacter oxydans* in Biofuel Cells

**DOI:** 10.3390/polym15051296

**Published:** 2023-03-03

**Authors:** Veronika Fedina, Daria Lavrova, Tatyana Dyachkova, Anastasia Pasko, Anton Zvonarev, Victor Panfilov, Olga Ponamoreva, Sergey Alferov

**Affiliations:** 1 Laboratory of Ecological and Medical Biotechnology, Tula State University, Friedrich Engels Street 157, 300012 Tula, Russia; 2 Biotechnology Department, Tula State University, Pr. Lenina 92, 300012 Tula, Russia; 3 Department of Technology and Methods of Nanoproducts Manufacturing, Tambov State Technical University, 106/5, Building 2, Sovetskaya Str., 392000 Tambov, Russia; 4 G.K. Skryabin Institute of Biochemistry and Physiology of Microorganisms, Federal Research Center “Pushchino Scientific Centre of Biological Research”, Russian Academy of Sciences, 142290 Pushchino, Russia; 5 Department of Biotechnology, Mendeleev University of Chemical Technology of Russia, Miusskaya Square 9, Moscow 125047, Russia

**Keywords:** electroactive polymer composites, chitosan, poly(vinyl alcohol), bovine serum albumin, membrane fraction of bacteria *Gluconobacter oxydans*, carbon nanotubes, immobilization

## Abstract

The development of biofuel cells (BFCs) currently has high potential since these devices can be used as alternative energy sources. This work studies promising materials for biomaterial immobilization in bioelectrochemical devices based on a comparative analysis of the energy characteristics (generated potential, internal resistance, power) of biofuel cells. Bioanodes are formed by the immobilization of membrane-bound enzyme systems of *Gluconobacter oxydans* VKM V-1280 bacteria containing pyrroloquinolinquinone-dependent dehydrogenases into hydrogels of polymer-based composites with carbon nanotubes. Natural and synthetic polymers are used as matrices, and multi-walled carbon nanotubes oxidized in hydrogen peroxide vapor (MWCNTox) are used as fillers. The intensity ratio of two characteristic peaks associated with the presence of atoms C in the sp^3^ and sp^2^ hybridization for the pristine and oxidized materials is 0.933 and 0.766, respectively. This proves a reduced degree of MWCNTox defectiveness compared to the pristine nanotubes. MWCNTox in the bioanode composites significantly improve the energy characteristics of the BFCs. Chitosan hydrogel in composition with MWCNTox is the most promising material for biocatalyst immobilization for the development of bioelectrochemical systems. The maximum power density was 1.39 × 10^−5^ W/mm^2^, which is 2 times higher than the power of BFCs based on other polymer nanocomposites.

## 1. Introduction

Currently, there is intensive progress in biotechnology associated with the development of biofuel cells as alternative sources of renewable energy. This is due to humanity’s understanding of global environmental problems that need to be solved to reduce the role of economic levers in world community development. According to PubMed (NCBI), there has been an exponential increase in publications on biofuel cells (BFCs) in the first decade of this century, and this concern has persisted throughout the following years. Biofuel cells based on immobilized microorganisms (microbial fuel cells (MFCs)) are promising for solving environmental problems due to their unique ability to generate energy and purify wastewater simultaneously. Some recent reviews summarize information on various aspects of MFC operation [1,2,3,4,5]. The methods of biocatalyst immobilization on the surface of electrodes are noted to play an important role in the efficiency of biofuel cells.

Significant progress in the development of nanotechnology, nanomaterials, and conductive polymer composites has allowed us to reach a new level of bioelectrocatalysis research to create highly sensitive biosensors [6,7,8,9] and miniaturized energy sources (BFCs) [10,11,12]. Redox-conducting or electroactive hydrogels based on composites of natural or synthetic polymers and redox compounds or carbon nanomaterials are used to organize extracellular electron transfer from enzyme systems of microorganisms [13,14,15]. Such composites typically use polymers that may not be electrically conductive but are capable of forming hydrogels. Electroactive compounds or conductive nanomaterials are a filler for the polymer matrix, and thus provide the electrical conductivity of the hydrogel in the composite [16,17]. The mechanism of electron transport through the hydrogels of electrically conductive polymer composites is still the subject of some fundamental research. The redox activity in such systems is assumed to arise from a series of successive redox interactions between closely spaced electroactive sites in the composite. Chitosan, bovine serum albumin, and poly(vinyl alcohol) (PVA) are used as polymers that initially do not have electrical activity but are capable of forming biocompatible hydrogels [18,19,20].

Chitosan, a natural cationic polysaccharide, is obtained by the deacetylation of chitin. In the last few years, much attention has been paid to this polymer [21]. This material is used for the immobilization of microorganisms, including the development of bioelectrochemical systems [16], since the natural polymer does not interfere with the flow of nutrients and oxygen to the leaving cells and has a positive effect on the long-term stability of BFCs [22]. A promising matrix for the immobilization of microorganisms is the matrix of poly(vinyl alcohol) modified with N-vinylpyrrolidone (PVA-VP) [20]. Previously, the possibility of developing BFCs based on the whole bacterial cells of *Gluconobacter oxydans* immobilized in a chemically modified PVA was shown. The energy characteristics of the obtained BFC model were significantly higher than similar BFC values based on a suspension of *G. oxydans* bacteria [23]. Bovine serum albumin (BSA) is also used as a biocompatible and biodegradable matrix for electrically conductive hydrogels [19].

Various approaches, including modification with carbon nanomaterials, are used to make polymer hydrogels conductive. Fibrous carbon materials, which include carbon nanotubes (CNTs) and nanofibers, are one of the most promising materials for the formation of conductive matrices [16]. CNTs have been shown to be particularly suitable for establishing electronic bonds with the active sites of enzymes, since they have a diameter from one to several tens of nanometers; thus, biocatalysts are allowed to carry out effectively either direct electron transfer to the electrode or are mediated by electronic shuttles that serve as intermediates for electron transfer [24,25,26,27]. Hydrogels based on polymer composites with nanomaterials significantly increase the energy characteristics of MFCs [28,29].

Bioelectrocatalytic systems use both single-walled carbon nanotubes (SWCNTs) and multi-walled carbon nanotubes (MWCNTs). MWCNTs are more attractive because of their low cost and stability in oxidative modification processes. Oxidative functionalization of CNTs increases their surface affinity to various solvents, polymeric materials, and biomolecules [30,31]. It should be noted that the information about the effect of oxidation on the electrically conductive properties of CNTs is contradictory. On the one hand, the violation of the integrity of graphite layers during the formation of functional groups should lead to a decrease in electrical conductivity. On the other hand, the opposite effects are often reported. Various explanations are proposed for the interpretation of the observed effects. The formation of interlayer bonds between carbon atoms in the state of sp^3^ hybridization facilitates the electron transition to the inner layers, the appearance of additional conduction bands near the Fermi level in the presence of oxygen-containing groups, and the formation of an integral system of initially separated CNTs [32,33]. Moreover, functionalized CNTs interact easily with polymer molecules, which leads to their uniform distribution in composites and an increase in the electrical conductivity of the material [34,35]. Thus, the method of oxidative modification of MWCNTs is important for obtaining conductive composites based on them [32], and it must be considered while developing polymer-based nanocomposites for bioelectrocatalysis.

One of the ways to develop miniature biofuel cells is the use of enzymatic cascades on a bioanode [36,37]. Enzyme cascades are numerous in nature, but they also have the potential for artificial applications due to the possibility of using various substrates in biofuel cells. Enzyme cascade reactions are distinguished by an expanded set of substrates, reaction depth, and increased overall performance. Fragments of bacterial membranes with localized enzymes can act as natural enzymatic cascades. Acetic acid bacteria of the genus *Gluconobacter* are microorganisms with a special organization of membrane-bound enzyme systems. These Gram-negative and obligate aerobes have an exceptional ability to perform regioselective oxidation of sugars, polyols, and alcohols with high productivity [38,39]. The substrate oxidation by these bacteria is carried out mainly by membrane-bound dehydrogenases (mDHs) localized on the outer side of the cytoplasmic membrane. These dehydrogenases are quinoproteins and flavoproteins and contain pyrroloquinoline quinone (PQQ) as a cofactor [40]. There are no enzymatic pathways of PQQ biosynthesis in most standard bacterial heterologous cloning systems, such as *E. coli*, so the creation of artificial multi-enzyme systems based on PQQ-DHs is difficult. It should be noted that mDHs are associated with the bacterial respiratory chain, the redox components of which are localized in the cytoplasmic membrane of bacteria [41,42,43,44].

Enzymes isolated from acetic acid bacteria and whole bacterial cells were used to develop biosensors [38,39,40,41,42,43,44,45] and BFCs [11,18,45]. The results of the relevant studies are summarized in previous reviews [13,46,47]. Thus, the membrane fractions of acetic acid bacteria can be considered as natural multi-enzymatic systems for the efficient oxidation of sugars, polyols, and alcohols.

The aim of this work is to select promising materials in bioelectrochemical devices by comparing the energy characteristics of biofuel cells with a bioanode based on conductive polymer composites and an immobilized biocatalyst—membrane-bound enzyme systems (membrane fractions) of acetic acid bacteria *Gluconobacter oxydans*.

## 2. Materials and Methods

### 2.1. Reagents and Materials

All chemicals were of analytical grade and used without further purification. d(+)Glucose, sorbitol, yeast extract (for use in microbial growth medium), agar powder bacteriological, chitosan (low molecular weight), poly(vinyl alcohol) (molecular weight, 1 × 10^5^–1.1 × 10^5^ AWU), N-vinylpyrrolidone, nitric acid, potassium hexacyanoferrate (III) (K_3_[Fe(CN)_6_]), 2,6-dichlorophenolindophenol (2,6-DCPIP), sodium hydrophosphate, sodium dihydrogen phosphate, and sodium dithionite were purchased from Sigma–Aldrich Chemicals (Steinheim, Germany). All aqueous solutions were prepared using water purified and deionized (18 MΩ) with an Aqualab AL-1 Double system (Aqualab Ltd., Moscow, Russia). PVA was modified with N-vinylpyrrolidone according to [20]. Multi-walled carbon nanotubes were kindly provided by Nanotech Center Ltd. (Tambov, Russia).

### 2.2. Cultivation of Microorganisms and Obtaining the Membrane Fraction of Bacteria Gluconobacter oxydans

The bacteria *Gluconobacter oxydans* VKM B-1280 used in the study were obtained from the All-Russian Collection of Microorganisms, FSBIS G.K. Skryabin Institute of Biochemistry and Physiology of Microorganisms, Russian Academy of Sciences. Bacteria were stored on a solid medium of composition: sorbitol, agar, and yeast extract. The inoculum was grown in a BIOSAN ES-20/60 shaker-incubator (BioSan, Riga, Latvia) on a liquid medium containing 200 g/L sorbitol and 20 g/L yeast extract for 24 h. A 15 mL volume of *G. oxydans* cell culture was used as inoculum in a flask with 150 mL of medium and grown in a shaker-incubator for 18 h. The resulting biomass was centrifuged in an MPW-351R centrifuge (MPW MED. INSTRUMENTS, Warsaw, Poland) at 4 °C for 10 min (10,000× *g*). The centrifugate was washed twice with phosphate buffer solution (pH = 6.0) for 10 min.

The precipitated cells were resuspended in fresh buffer solution and disrupted on a “Qsonica (Q125)” ultrasonic processor (Qsonica L.L.C., Newtown, CT, USA) for 40 min at an amplitude of 80%. Then, the disrupted cells were centrifuged at 4 °C for 40 min (5000× *g*) to precipitate large cell debris. The resulting lysate was centrifuged in a Beckman Coulter Avanti J-30I centrifuge (Beckman Coulter Life Sciences, Brea, CA, USA) at 4 °C for 40 min (101,000× *g*) to precipitate membrane fractions of *Gluconobacter oxydans* cells. The membrane fraction was stored at −18 °C.

### 2.3. Dehydrogenase Activity of the Membrane Fraction

The measurements were carried out using an SF-2000 spectrophotometer (OKB Spektr, St. Petersburg, Russia) in the kinetic mode at a wavelength of 600 nm. The quinoneimine dye 2,6-dichlorophenolindophenol was used to determine the activity of membrane enzymes; the concentration in the measuring cuvette was 0.6 mM. A 160 µL volume of 2,6-DCPIP, 773 µL of phosphate buffer solution (pH = 6.0), and 10 µL of *G. oxydans* enzyme fraction were injected into the reference cuvette. A 160 µL volume of 2,6-DCPIP mediator, 780 µL of phosphate buffer solution (pH = 6.0), and 10 µL of *G. oxydans* enzyme preparation were injected into the measuring cuvette. Then, the solutions were thoroughly mixed. A 57 μL volume of 15% sodium dithionite solution was added to the reference cuvette to reduce 2,6-DCPIP, and 50 μL of 1 M glucose solution (final concentration 50 mM) was supplemented to the measuring cuvette. The solutions in both cuvettes were thoroughly mixed over, and the dependences of the optical density on time were taken for 600 s.

A unit of enzymatic activity is the amount of enzyme capable of causing the conversion of 1 µmol of substrate per minute at 25 °C under optimal conditions. The specific activity of an enzyme is the number of units of enzymatic activity per 1 mg of protein. The specific activity is, respectively, expressed in µmol/(mg × min) (formula 1).

(1) $$A_{spec}=\frac{tg\alpha \times V}{\varepsilon \times l\times m_{prot}{}_{\;}}$$

where *A_spec_* is the specific activity (µmol/mg × min), *tgα* is the reduction rate constant of the redox dye (s^−1^), *V* is the volume of the solution in the cuvette (l), *ε* = 21,300 is the molar absorption coefficient of 2,6-DCPIP (*l*/(mol × cm), *l* is the thickness of the absorbing layer (1 cm), and *m_prot_* is the amount of protein (g).

### 2.4. Preparation of Graphite Electrodes and Carbon Nanotubes

#### 2.4.1. Treatment of Graphite Electrodes with Concentrated Nitric Acid to Give Them a Microrelief

Spectral graphite rods with a diameter of 8 mm (JSC NIIEI, Elektrougli, Russia) were used as working electrodes. The preparation of graphite electrodes was carried out according to the method presented in [23].

#### 2.4.2. Synthesis and Oxidation of Multi-Walled Carbon Nanotubes

Multi-walled carbon nanotubes (MWCNTs) were obtained at 630 °C from a propane–butane mixture with a Co/Mo/Mg/Al catalyst [47]. MWCNTs were oxidized in 37% hydrogen peroxide vapor at 120 °C for 10 h according to the procedure described in [45]. X-ray photoelectron spectroscopy was used to determine the content and chemical state of oxygen and carbon atoms in oxidized MWCNTs. The spectra were recorded on an Axis Ultra DLD spectrometer (Kratos Analytical, Manchester, UK) using monochromatic Al K_α_ radiation (1486.7 eV). Survey XPS spectra were recorded at a pass energy of 160 eV, while a pass energy of 40 eV was used for high-resolution scans. Identification of surface functional groups formed during CNT oxidation was carried out by FTIR spectroscopy on the Jasco FT/IR 6700 instrument (JASCO Corporation, Tokyo, Japan) in the wave range of 4000–550 cm^−1^.

### 2.5. Scanning Electron Microscopy for Redox-Active Polymers

The surface morphology of the sample was examined by scanning electron microscopy (SEM). Samples of the initial polymers, polymers with oxidized MWCNTs, and polymers with oxidized MWCNTs and membrane fraction of *G. oxydans* were fixed at 4 °C for 12 h in 0.05 M sodium cacodylate buffer (pH 6.8) containing 1.5% glutaraldehyde and then post-fixed at 20 °C for 3 h in the same buffer supplied with 1% OsO_4_. The samples were coated with gold after dehydration (Fine Coat Ion Sputter JFC-1100, Tokyo, Japan) and examined with a scanning microscope JSM-6510LV (JEOL, Tokyo, Japan).

### 2.6. Biofuel Cell Setup

**Figure 1:**
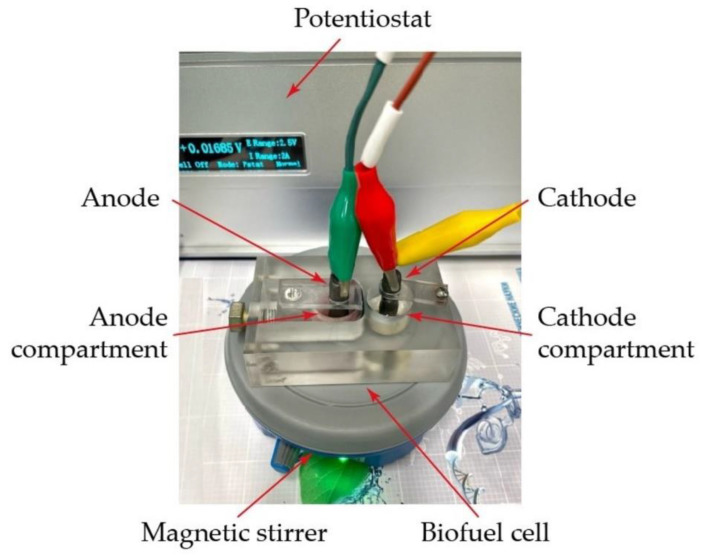
Microbial fuel cell used in this study.

The measurements were carried out using an electrochemical station CS "CORRTEST" series (Corrtest Instruments, Wuhan City, China). The biofuel cell consisted of two interconnected chambers; the volume of the anode compartment equaled the volume of the cathode compartment and was 3 mL (Figure 1). Spectral graphite rods with a diameter of 8 were used as electrodes. An electrode without a biocatalyst was placed in the cathode compartment, and an electrode with membrane fractions of *G. oxydans* bacteria immobilized in different matrices was placed in the anode compartment; the depth of electrode immersion in the solution was 10 mm. A 30 mM sodium phosphate buffer solution with pH = 6.0 was used as a working solution; 2,6-DCPIP in the anode chamber and K_3_[Fe(CN)_6_] in the cathode chamber were applied as redox compounds. The modified electrodes were washed until the potential reached 0 mV, then redox compounds were brought, and when the steady state was established, the substrate was added to the anode compartment. The anode and cathode chambers were separated by a proton-selective MF-4SK membrane (Plastopolimer, Sankt-Petersburg, Russia) with a 6 mm diameter and an analog of the Nafion-117 membrane in the protonated form (Figure 1).

Resistances from 3 kΩ to 47 kΩ were connected to the external circuit to determine the power characteristics. The maximum power of the biofuel cell is achieved in the case of equality of external and internal resistance. The generated potential of the closed and open circuit and the internal resistance of the BFC cell were determined by potentiometry. Other energy characteristics: maximum and power density were calculated by Formulas (2) and (3), respectively [48]:

P_max_ = (E^2^_MFC_)/R_ext_, (2)

P_dens_ = P_max_/S, (3)

where P is the BFC power, E is the generated potential, R_ext_ is the external resistance, and S is the electrode surface area.

### 2.7. Formation of a Graphite Anode Based on Conductive Polymer Composites with Membrane Fractions of Gluconobacter oxydans Cells

Membrane fractions of *Gluconobacter oxydans* bacteria were immobilized using two approaches: (a) while the biocatalyst suspension was applied directly to a graphite electrode and fixed with a conductive polymer; (b) the membrane fraction was included in a conductive polymer composite and applied to the electrode.

#### 2.7.1. Preparation of a Conductive Polymer Composite Based on Chitosan

Chitosan was mixed with 100 μL of 1% acetic acid and stirred in a CM-70M-07 centrifuge-vortex (ELMI, Riga, Latvia) for 3 min to make a conductive matrix (2.9 × 10^−8^ mol). T = 0.22 μg/μL of carbon nanotubes was added and stirred for 1 min. Then, 1.4 × 10^−7^ mol of glutaraldehyde was brought, and the mixture was shaken for 30 s. The resulting mixture was applied to a graphite electrode (application height 10 mm) and left at a temperature of 5 °C until drying (18 h) [46].

#### 2.7.2. Preparation of a Conductive Polymer Composite on Poly(vinyl alcohol) Modified with N-vinylpyrrolidone

To prepare an electroactive matrix, 100 μL of PVA modified with N-vinylpyrrolidone (the modification was carried out according to [20], and the proposed mechanism is presented in Supplementary Materials Figure S1) was mixed with T = 0.22 μg/μL of carbon nanotubes, stirred for 2 min, deposited on a graphite electrode, and left at 5 °C until completely drying (18 h).

#### 2.7.3. Preparation of Polymeric Conductive Composite Based on Bovine Serum Albumin

A 0.0035 g (5 × 10^−8^ mol) amount of BSA was weighed (the quantity of amino groups in BSA 4.13 × 10^−9^ mol) and dissolved in 50 μL of phosphate buffer solution (pH = 6.0). After dissolution, T = 0.22 μg/μL of carbon nanotubes and stirred for 3 min. Then, 8 × 10^−8^ mol of glutaraldehyde was added, stirred for 30 s, and applied to the surface of a graphite electrode. The modified electrode was left at a temperature of 5 °C until completely dry (18 h).

## 3. Results and Discussion

### 3.1. Characterization of Membrane Fractions of Acetic Acid Bacteria by Dehydrogenase Activity

**Figure 2:**
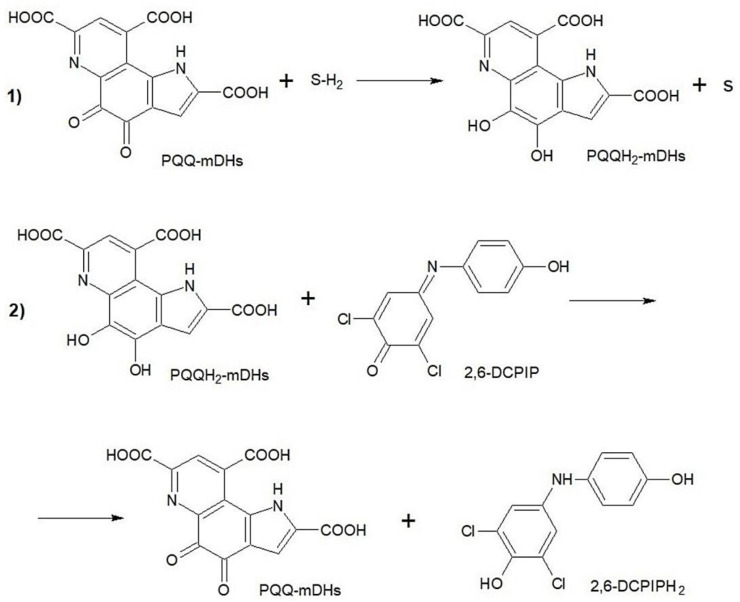
2,6-DCPIP interaction with PQQ-mDHs of *G. oxydans*: 1. Charge transfer from the substrate to the active site of the enzyme; 2. Charge transfer from an enzyme to an artificial electron acceptor. SH_2_ is the reduced form of the substrate (glucose); S is the oxidized form of the substrate (glucono-1,5-lactone).

As mentioned above, membrane DH is involved in the oxidation of most carbohydrates and alcohols in acetic acid bacteria *G. oxydans*, which determines the biocatalytic properties of their membrane fractions. Methods for determining the activity of many oxidoreductases are based on their ability to reduce redox dyes to the leucoform (colorless compounds) in the presence of substrates, which is recorded spectrophotometrically. To determine the activity of quino- and flavoproteins, the quinoneimine dye 2,6-dichlorophenolindophenol is used. This reagent is able to change color depending on the pH of the medium and the oxidation state [47]. In [49], it was proposed to use 2,6-DCPIP for the detection of viable microorganisms. The absorbance of 2,6-DCPIP decreased at 600 nm due to the dye reduction by microbial membrane enzymes and was inversely proportional to the number of viable cells. However, this method is based on the determination of the dehydrogenase activity of membrane-localized enzymes (Figure 2); therefore, this method was used to characterize the membrane fractions of acetic acid bacteria.

The dehydrogenase activity in 1 g of the membrane fraction was 69 ± 8 μmol/min. The obtained activity value is comparable with the values obtained when determining the membrane-bound dehydrogenase of whole bacteria cells of *Gluconobacter oxydans* by the same method in the biotechnological production of 2-keto-gluconic acid [50]. Thus, the membrane fractions of acetic acid bacteria obtained by the described method have a high dehydrogenase activity and can be used as a biocatalyst on the BFC anode. This will reduce the role of physiological aspects of the activity of living cells and will allow using only the catalytic activity of enzyme systems of microorganisms.

### 3.2. Characterization of Multi-Walled Carbon Nanotubes as a Filler for Conductive Polymer-Based Nanocomposites

MWCNTs were used as fillers, and they were obtained in CVD synthesis from a propane–butane mixture in the presence of metal oxide (Co/Mo/Mg/Al) catalysts and oxidized in hydrogen peroxide vapor (MWCNTox). Oxidation in hydrogen peroxide vapor provides the formation of oxygen-containing groups on the MWCNTs’ surface without increasing the defect degree [45].

**Figure 3:**
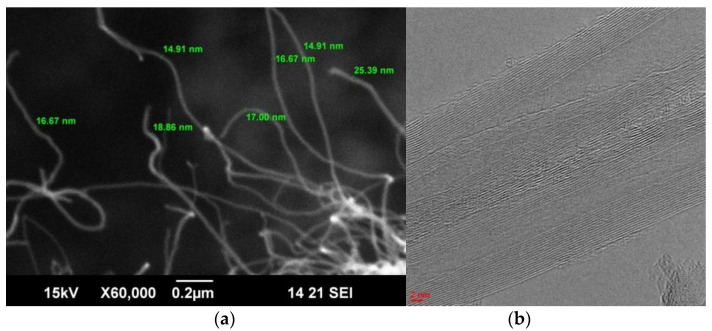
SEM (**a**) and TEM (**b**) images of MWCNTox.

SEM and TEM images of MWCNTox are shown in Figure 3. The outer diameter of the nanotubes varies in the range of 15–30 nm. The diameter of the inner channel is 4–8 nm, the length is more than 2 μm, and the number of graphene layers varies from 8 to 20. The specific surface area (according to the BET analysis) is 260 m^2^/g.

**Figure 4:**
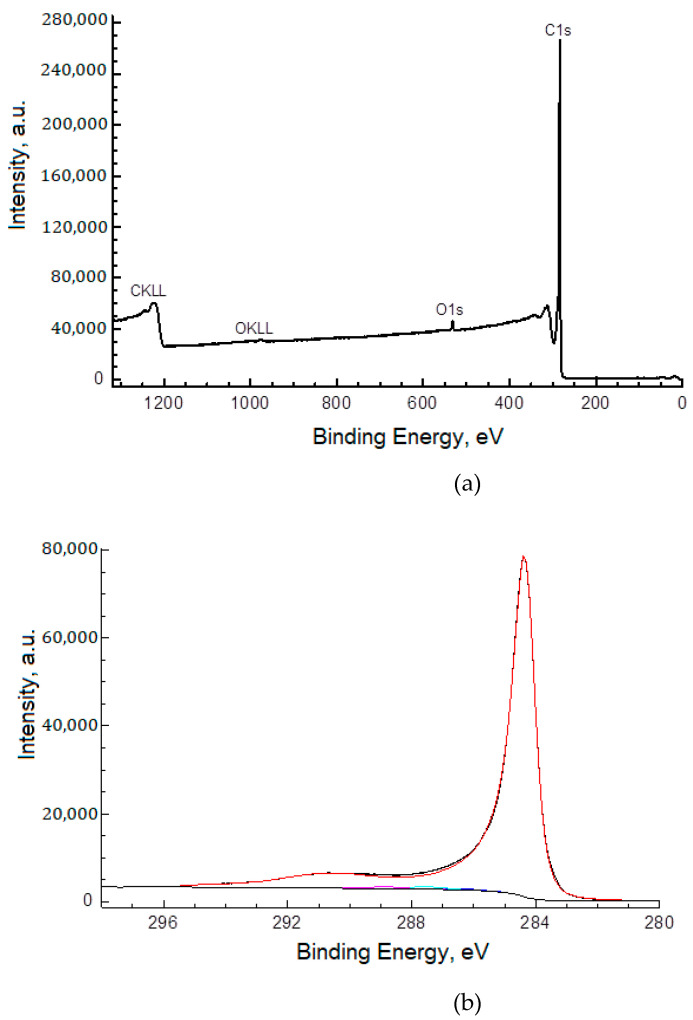

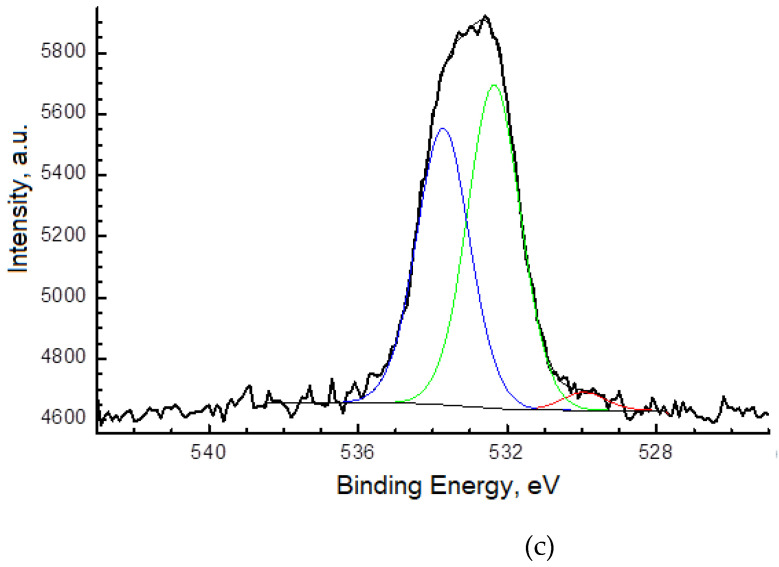
XPS spectra of MWCNTox (**a**) survey spectrum, (**b**) C 1s spectrum, and (**c**) O1s spectrum.

Based on the analysis of X-ray photoelectron spectra (Figure 4) of MWCNTox, the content of elements, binding energies, and fractions of components on their surface were determined (Table 1). The carbon material contains about 1.1 atomic% of oxygen in the form of O_2_–, O=C, and O–C.

**Figure 5:**
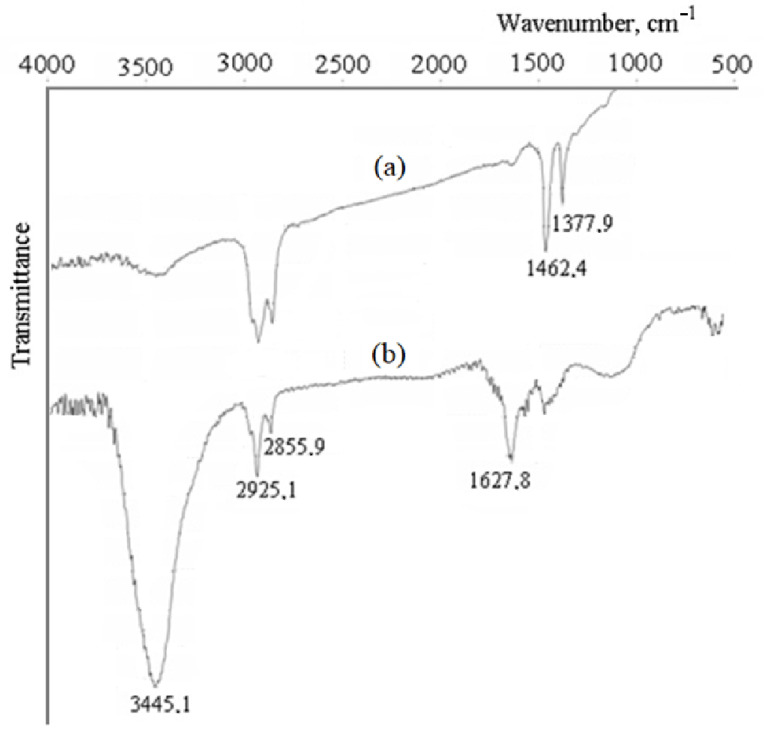
FTIR spectra of the pristine (**a**) and oxidized (**b**) MWCNTs.

IR spectra provide information about changes in the chemical composition of the MWCNTs surface after oxidation in hydrogen peroxide vapor (Figure 5).

Only bands corresponding to asymmetric (2925 cm^−1^) and symmetric (2856 cm^−1^) stretching and deformation fluctuations (1462 and 1378 cm^−1^) of the C-H bonds in alkyl groups are usually observed in the initial samples (Figure 5a) [51]. These groups are related to the fragments of hydrocarbon molecules, on the basis of which CNTs are obtained by the CVD method [52]. The oxidation leads to a strong decrease in the intensity characteristic of C-H bonds (Figure 5b), whereas an intense peak attributed to fluctuations of O-H bonds in hydroxyl appears at 3445 cm^−1^. Moreover, the peak (maximum absorption) occurs at 1628 cm^−1^; it can be associated with fluctuations of the C=O bonds in the group > C=O [53].

**Figure 6:**
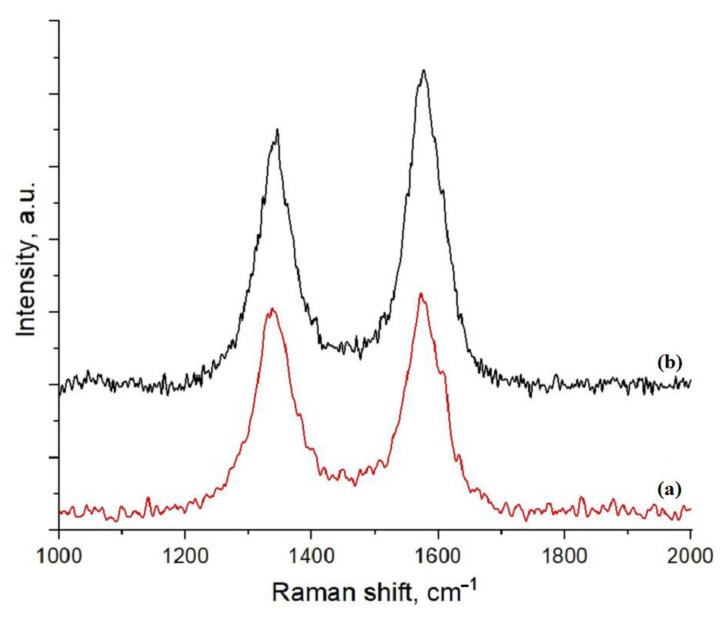
Raman spectra of pristine (**a**) and oxidized (**b**) MWCNTs.

A distinctive feature of MWCNTs oxidized in hydrogen peroxide vapor is a reduced degree of defectiveness compared to the pristine nanotubes. It was estimated from the data of Raman spectra (Figure 6). Two characteristic peaks are observed: G (~1570 cm^−1^), caused by fluctuations of carbon atoms in the sp^2^ hybridization in the plane of the graphene layer, and D (~1350 cm^−1^), associated with the presence of atoms in the sp^3^ hybridization and, as a consequence, with a violation of the symmetry of ideal graphene layers [54]. The intensity ratio of the i_D_/i_G_ bands for the pristine and oxidized materials is 0.933 and 0.766, respectively.

A decrease in the defect index value provides an increase in the electrical conductivity of nanotubes [55]. We assume that the oxidation of MWCNTs in hydrogen peroxide vapors allowed, on the one hand, to functionalize the initial CNTs for better interaction with the polymer matrix and enzymes, and, on the other hand, to increase the electrical conductivity of polymer-based nanocomposites by using oxidized MWCNTs as a filler.

### 3.3. Scanning Electron Microscopy of Conductive Polymer-Based Composite

**Figure 7:**
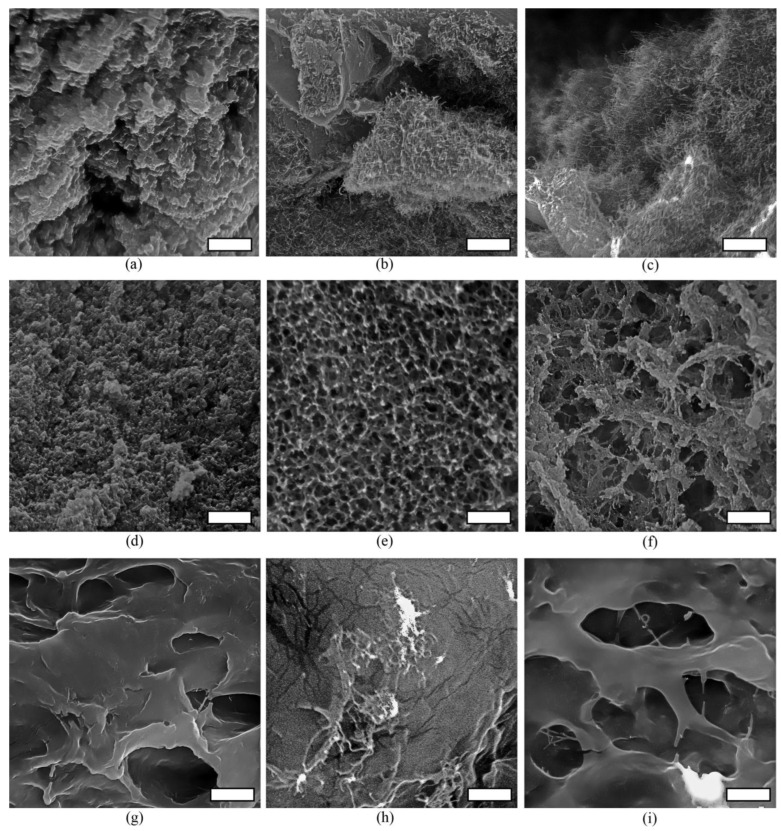
Scanning electron microscopy images (scale bar is 2 µm for all images) of conductive polymer-based composites: (**a**) initial chitosan hydrogel, (**b**) chitosan/MWCNTox composite, (**c**) chitosan/MWCNTox/membrane fraction of *G. oxydans*, (**d**) initial BSA, (**e**) BSA/MWCNTox composite, (**f**) BSA/MWCNTox/membrane fraction of *G. oxydans*, (**g**) initial PVA-VP hydrogel, (**h**) PVA-VP/MWCNTox composite, and (**i**) PVA-VP/MWCNTox/membrane fraction of *G. oxydans*.

The SEM method was used to characterize conductive polymer-based composites. The resulting images are shown in Figure 7.

The structure of polymer matrices and polymer-based nanocomposites was studied by SEM. Polymer matrices, based on natural chitosan and BSA compounds, form homogeneous gels (Figure 7a,d), while the synthetic polymer PVA-VP forms hydrogels with a pore size of 3–10 microns (Figure 7g). In the presence of MWCNTox, a structure with an orderly uniform distribution of nanotubes over the entire volume of the nanocomposite is formed in the chitosan hydrogel (Figure 7b). Recently, Jin and co-authors synthesized a CNTs/chitosan composite to remove organic dyes [56] and showed that the adsorption of chitosan on the surface of carbon nanotubes improves the interaction of redox dyes with CNTs. This suggests the possibility of effective contact between redox compounds, an electrically conductive matrix, and enzymes. The presence of bacterial membrane structures has no significant effect on the nanocomposite (Figure 7c).

The addition of MWCNTox to the BSA leads to the formation of an ordered mesh structure with a pore diameter of less than 1 micron (Figure 7e). At the same time, carbon nanotubes are not visualized in a free form, as in Figure 7b,c. We suggest that protein packaging is formed on the surface of CNTs within the conditions of composites’ synthesis. The interaction of BSA with carboxylated SWCNTs [57] and carboxylated MWCNTs [58] has been previously investigated, and it has been shown that protein agglomerates form on the surface of nanotubes. It is noted that the key role in the formation of such structures is played by hydrophobic interactions between the graphene surface of carbon nanotubes and protein sites, with a high density of hydrophobic residues. At the same time, the micrographs, obtained in [59] by the SEM method of SWCNTs samples in BSA cross-linked with glutaraldehyde, clearly distinguish separate SWCNTs strands in the protein matrix. The formation of the protein packaging of nanotubes may be due to the ability of oxidized MWCNTs to form hydrophobic–hydrophilic frameworks in an aqueous medium for a special interaction with proteins. A similar behavior of MWCNTs has been demonstrated in their interaction with thermoplastic polyurethane [60]. It was shown that MWCNTs act as nucleating components for the hard segment formation of TPU after the annealing process. When bacterial membrane structures are added to the BSA-MWCNTox composite, the ordered structure is largely destabilized, and pores from 2 to 5 microns are formed (Figure 7f). The presence of diphilic membrane lipids leads to the disruption of hydrophobic interactions between the protein and MWCNTox. Nevertheless, it can be expected that protein shells on the CNT surface in nanocomposites based on BSA-MWCNTox will prevent the effective transfer of electrons from the active centers of enzymes with the participation of MWCNTox.

In the PVA-VP hydrogel, the distribution of nanotubes is uneven, with the obvious formation of conglomerates in several places (Figure 7h). This is due to the high hydrophilicity of the polymer matrix in the absence of cationic groups in the polymer for electrostatic interaction with MWCNTox. The hydrogel structure, whose pore diameter is 2-5 microns, is preserved when the membrane fractions are added subsequently (Figure 7i). Single nanotubes are visualized in some areas of bionanocomposite structure. We suggest that the uneven distribution of the electrically conductive material in the polymer matrix will interfere with the effective functioning of the BFC. Thus, a composite based on MWCNTox and chitosan should demonstrate the best electroconducting properties in the studied BFC models.

### 3.4. Biofuel Cell Functioning with a Bioanode Based on Natural Enzymatic Cascades Immobilized into Polymer-Based Composites 

**Figure 8:**
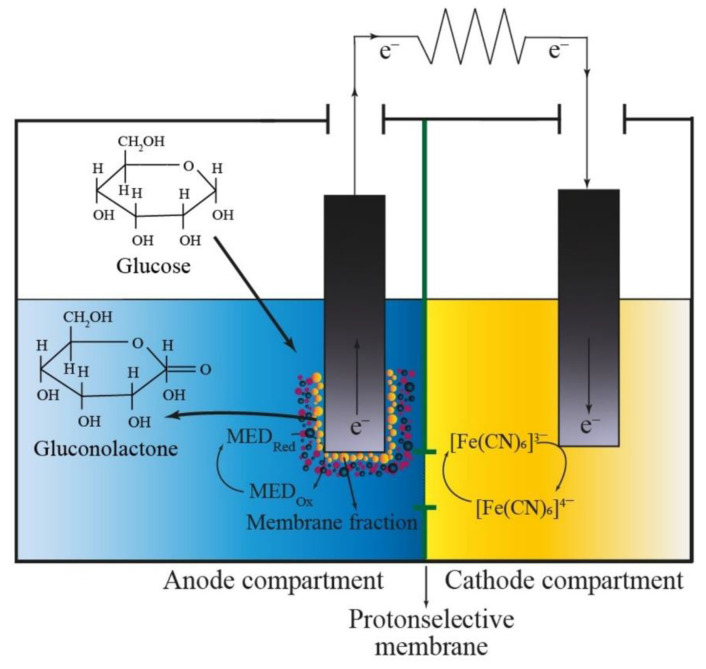
Operation of a microbial fuel cell.

A comparative analysis of the BFC characteristics determined the choice of polymer-based composites in combination with oxidized MWCNTs as promising materials for the development of bioelectrochemical devices. Composites filled with oxidized MWCNTs based on chitosan, PVA modified with N-vinylpyrrolidone, and cross-linked BSA were used to immobilize natural cascades of enzymes (membrane fractions of acetic acid bacteria *Gluconobacter oxydans*) on the surface of graphite electrodes. The mode of BFC operation, used in this work, is shown in Figure 8.

Further electron transfer is possible directly to the 2,6-DCPIP artificial acceptor when glucose is oxidized by PQQ dehydrogenases. When the external circuit is closed, the reduced 2,6-DCPIP is oxidized at the anode, and the electrons from the anode move to the cathode through the external load. K_3_[Fe(CN)_6_] is reduced at the cathode. The flow of electrons through an external load causes an electric current.

**Figure 9:**
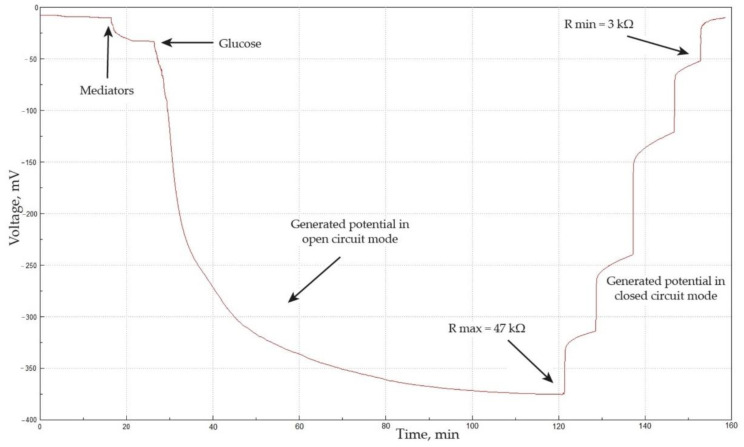
Experimental dependence of the generated potential to determine the parameters of the functioning of the BFC (a membrane fraction is layer-by-layer immobilized on the anode, fixed by a conductive polymer composite based on chitosan).

A typical experimental dependence for determining the parameters of a biofuel element is shown in Figure 9.

The MFC potential is about 0 mV in the absence of redox compounds in the anode and cathode chambers. An increase in the potential is observed when 2,6-DCPIP and K_3_[Fe(CN)_6_] are added into the system, which is due to redox processes at the anode and cathode. 2,6-DCPIP, as a mediator of electron transfer from membrane-bound enzymes of acetic acid bacteria to the electrode [57], is partially reduced even in the absence of substrates, which leads to a change in the concentration ratio of 2,6-DCPIPox/2,6-DCPIPred in the near-electrode area and to a change in the potential at the bioanode respectively. When glucose is added to the anode chamber, the generated potential increases significantly in the open-circuit mode due to the enzymatic oxidation of glucose and the interrelated enzymatic reduction of 2,6-DCPIP. This process can be considered as a two-substrate enzymatic reaction, as has been shown earlier in the whole cells of acetic acid bacteria [61]. The potential reaches a steady state in the open-circuit mode, which is due to some equilibrium when all 2,6-DCPIP molecules are reduced in the near-electrode area. An abrupt decrease in potential is observed with the subsequent connection of resistances of 47 kΩ, 30 kΩ, 13 kΩ, 5 kΩ, and 3 kΩ, which indicates the reoxidation of 2,6-DCPIP at the anode and the reduction of K_3_[Fe(CN)_6_] at the cathode. Thus, each time a new equilibrium is established in the bioelectrochemical system with a different external load in the circuit, which leads to a certain potential. The value of this potential depends on the ratio of the rates of various electrochemical stages (enzymatic reduction of 2,6-DCPIP, diffusion of the mediator to the electrode, electrochemical oxidation of 2,6-DCPIP, and, finally, reduction of K_3_[Fe(CN)_6_] at the cathode).

### 3.5. Comparative Evaluation of the BFC Energy Parameters with Joint Deposition of Conductive Polymer-Based Nanocomposites and Membrane Fraction of G. oxydans Cells

The energy characteristics of the MFC using bioanodes based on biocomposites (immobilized biomaterial in polymer-based nanocomposites) are presented in Table 2. The dependence of power on external load is presented in Supplementary Materials Figure S2.

**Figure 10:**
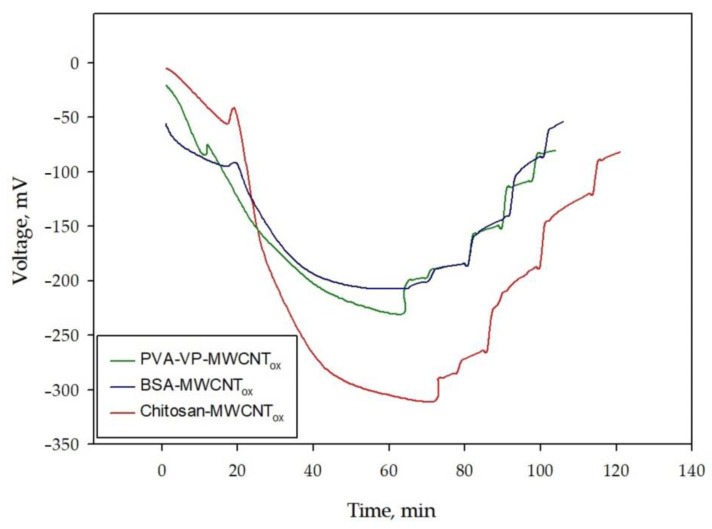
Experimental polarization curves of BFC with bionodes based on various composites with biomaterial immobilization in polymer-based nanocomposites.

A comparison of the experimental polarization curves of the BFC with anodes based on various conductive polymer composites (immobilized biomaterial in polymer-based nanocomposites) is presented in Figure 10.

The highest energy characteristics of the BFC were obtained using a composite hydrogel based on a chitosan: the power density reached 1.05 × 10^−5^ W/mm^2^, and the maximum power (37 ± 4) × 10^−4^ W was achieved with an external resistance of 9.1 kΩ. The obtained results are explained by the uniform distribution of the biocatalyst in the chitosan-based matrix on the one hand and the more effective interaction of redox compounds with nanocomposite [55] on the other hand. It has been shown in [16] that the acetic acid bacteria *Gluconobacter oxydans* mixed with chitosan are evenly distributed over the conductive composite, and this state does not change over time. *G. oxydans* bacteria form small conglomerates in the PVA hydrogel modified with N-vinylpyrrolidone, and their size varies over time. The authors attribute the observed results to the adverse effect of this matrix on bacterial cells, and as a result, bacteria tend to minimize surface contact with the matrix. We have previously demonstrated that the self-organization of the *G. oxydans* cells in modified PVA hydrogel occurs by forming an extended network of bacterial cells aligned in a partial side-by-side configuration and producing biological clusters. This positioning is most likely set up to facilitate efficient electron transfer, as it results in an increased interaction contact area. The alignment of *G. oxydans* cells in the polymeric hydrogel allows the microbial fuel cells containing them to generate a greater electrical potential than that obtained by suspended *G. oxydans* cells in the absence of a hydrogel [62]. This effect is also possible to be observed when using the membrane structures of these cells.

The use of the BSA protein matrix in the composite does not lead to a significant increase in energy performance compared to the polymer composites mentioned above. Apparently, there is a rapid desorption of the biomaterial from the protein matrix. The authors of [19] note that for the best fixation of biocatalysts on electrodes or any other surfaces, bovine serum albumin is mostly used in combination with other polymer gels, leading to composite stabilization.

### 3.6. Comparative Evaluation of the MFC Energy Parameters with Layer-by-Layer Deposition of the Bacteria Membrane Fraction of G. oxydans and Polymer-Based Nanocomposites

The energy characteristics of the BFC using bioanodes based on layer-by-layer immobilization of biomaterial and polymer-based nanocomposites are shown in Table 3. The dependence of power on external load is presented in Supplementary Materials Figure S3.

**Figure 11:**
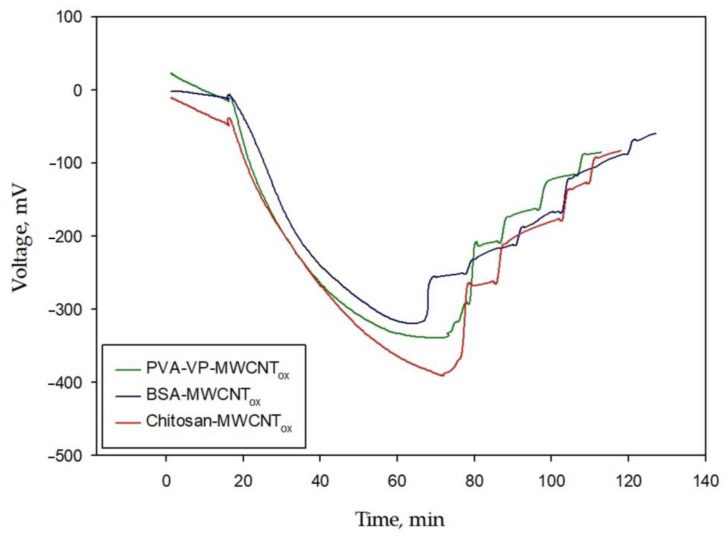
Experimental polarization curves of BFC with a bioanode based on various composites with layer-by-layer immobilization of biomaterial and composite.

A comparison of the experimental polarization curves of the BFC with a bioanode based on various conductive polymer composites during layer-by-layer immobilization of the biomaterial and composite is presented in Figure 11.

The energy characteristics of the MFC using layer-by-layer immobilization of the membrane fraction and the chitosan nanocomposite are higher than those of other systems. The main factor in the operation of the MFC is the power density. Earlier in [63], layer-by-layer immobilization of bacterial cells and chitosan matrix was carried out on thermally expanded graphite of a stencil electrode during the development of a biosensor. The obtained results allow us to conclude about the high sensitivity of the sensor and the feasibility of using devices with a similar configuration of a biocatalyst and an immobilizing matrix for BFC bioanodes.

### 3.7. Effect of Oxidized Nanotubes on the Potential Generation in BFCs

MWCNTs were not used in chitosan hydrogel in the preliminary experiments on the development of the BFC. The bioanode was formed by layer-by-layer immobilization of the membrane fraction of *G. oxydans* bacteria and the chitosan polymer matrix. The energy characteristics of such a BFC were less than the energy characteristics with MWCNTox. The generated potential in the closed-circuit mode was 234 ± 12 mV, and the power density reached 0.108 W/mm^2^ with the output power of the BFC model, (38 ± 2) × 10^−4^ W at a resistance of 11 kΩ. Thus, when using a chitosan-based polymer composite with the addition of oxidized MWCNTs, the power of the BFC increases significantly, but the internal resistance of the cell changes slightly. The work [64] studied the electrochemical properties of chitosan-based polymers in a composition with carboxylated nanotubes and PQQ-dependent glucose dehydrogenase in the anode compartment and bilirubin oxidase in the cathode one. Such a system demonstrated a maximum power of 1.56×10^−4^ W, and the power density was 0.16 W/m^2^. The BFC developed in this work allows to obtain 30 times higher power.

The authors of other studies note the important role of CNTs as a filler in composites mainly for the immobilization of enzymes. It has been shown in [65] that CNTs are an excellent background for the development of bioelectrocatalysts due to their large surface area, excellent electronic conductivity, and high chemical and structural stability. It has been found that the use of CNTs facilitates the electron transfer between biomaterials and the electrode surface. This leads to an increase in the efficiency of biocatalysts in biofuel cells and biosensors. In [66], glucose oxidase (GOx) was immobilized on an electrode in a chitosan matrix cross-linked with glutaraldehyde, in a composition with CNTs and a mediator to develop a glucometer. A comparative analysis of glucose determination using the developed biosensor and biosensors based on GOx electrodes without CNTs showed a significant increase in signals due to an increase in the electrical conductivity of the chitosan-based matrix with CNTs. Our results on the development of bioanodes based on bacterial membrane structures containing a pool of key enzymes for the oxidation of carbohydrates, polyols and alcohols, and chitosan nanocomposites with MWCNTs oxidized with hydrogen peroxide expand the possibilities of using such composites in the bioelectrocatalysis.

This study demonstrates the possibilities of using natural enzymatic cascades (membrane structures of acetic acid bacteria) immobilized with polymer-based nanocomposites for the development of BFCs. Oxidized under mild conditions, MWCNTs were used for the first time to ensure effective electron transfer from enzyme systems to the electrode with mediators. Such an approach for the MWCNTs modification made it possible to obtain a chitosan-based nanocomposite that facilitates the mediator-mediated electronic transfer from membrane-localized enzyme systems to the BFC anode.

## 4. Conclusions

Our study proves the possibilities of using natural enzymatic cascades (membrane structures of acid bacteria) immobilized with polymer-based nanocomposites for the development of BFCs. Oxidized under mild conditions MWCNTs were used for the first time to ensure effective electron transfer from enzyme systems to the electrode with the mediators. MWCNTox in a polymer composite increase the performance of BFCs, since they not only have conductive properties, but also an adsorbing ability in relation to redox compounds, which provides a higher concentration of the mediator in the near-electrode space. This approach for the modification of the MWCNTs has made it possible to obtain a chitosan-based nanocomposite facilitating mediator-mediated electronic transfer from membrane-localized enzyme systems to the BFC anode.

## Figures and Tables

**Table 1:** Contents of elements, binding energies, and fractions of components in XPS spectra, and the corresponding types of bonds for the studied sample.

Spectra	Element Content, at.%	Binding Energies, eV	Share, at.%	Binding Type
O1s	1.10	530.0	0.03	O^2–^
		532.4	0.58	O=C
		533.7	0.49	O–C
C1s	98.90	284.4	97.24	C–C (sp^2^)
		284.9	0.00	C–C,H (sp^3^)
		286.5	0.36	C–O
		287.5	0.74	C=O, O–C–O
		288.8	0.56	O=C–O

**Table 2:** Comparative evaluation of the MFC performance with joint deposition of conductive polymer composites with membrane fractions on the electrode *.

Polymer-Based Nanocomposite	E_closed circuit_^1^, mV	R_ext_^2^, kΩ	P ^3^ (×10^−4^), W	P_dens_^4^ (×10^−5^), W/mm^2^
Chitosan - MWCNTox	185 ± 3	9.1	37 ± 4	1.05
PVA-VP - MWCNTox	106 ± 4	5.1	22 ± 4	0.63
BSA- MWCNTox	137 ± 3	11.0	18 ± 4	0.51

^1^ E_closed circuit_ is the generated potential in closed-circuit mode; ^2^ R_ext_ is the external resistance; ^3^ P is the maximum power; ^4^ P_dens_ is the power density. * Data are given with confidence intervals at n = 5, with probability p = 0.95.

**Table 3:** Comparative evaluation of the BFC performance with layer-by-layer deposition of biomaterial and conductive polymer composite on the electrode *.

Polymer-Based Nanocomposite	E_closed circuit_^1^, mV	R_ext_^2^, kΩ	P ^3^ (×10^−4^), W	P_dens_^4^ (×10^−5^), W/mm^2^
Chitosan - MWCNTox	176 ± 9	10.0	49 ± 4	1.39
PVA-VP - MWCNTox	114 ± 3	5.1	26 ± 1	0.74
BSA - MWCNTox	164 ± 3	10.0	22 ± 2	0.63

^1^ E_closed circuit_ is the generated potential in closed-circuit mode; ^2^ R_ext_ is the external resistance; ^3^ P is the maximum power; ^4^ P_dens_ is the power density. * Data are given with confidence intervals at n = 5, with probability p = 0.95.

## Data Availability

Not applicable.

## Supplementary Materials

The following supporting information can be downloaded at: https://www.mdpi.com/article/10.3390/polym15051296/s1, Figure S1: The proposed mechanism of PVA modification with N-vinylpyrrolidone. Figure S2: The dependence of power on external load for BFC with joint deposition of conductive polymer composites with membrane fractions on the electrode. Figure S3: The dependence of power on external load for BFC with layer-by-layer deposition of biomaterial and conductive polymer composite on the electrode.
