# Supplementary figures and images for: Polymer-Based Conductive Nanocomposites for the Development of Bioanodes Using Membrane-Bound Enzyme Systems of Bacteria Gluconobacter oxydans in Biofuel Cells
Source: Polymers (Basel). 2023 Mar 3;15(5):1296. doi: 10.3390/polym15051296 (PMC10007125; doi:10.3390/polym15051296)

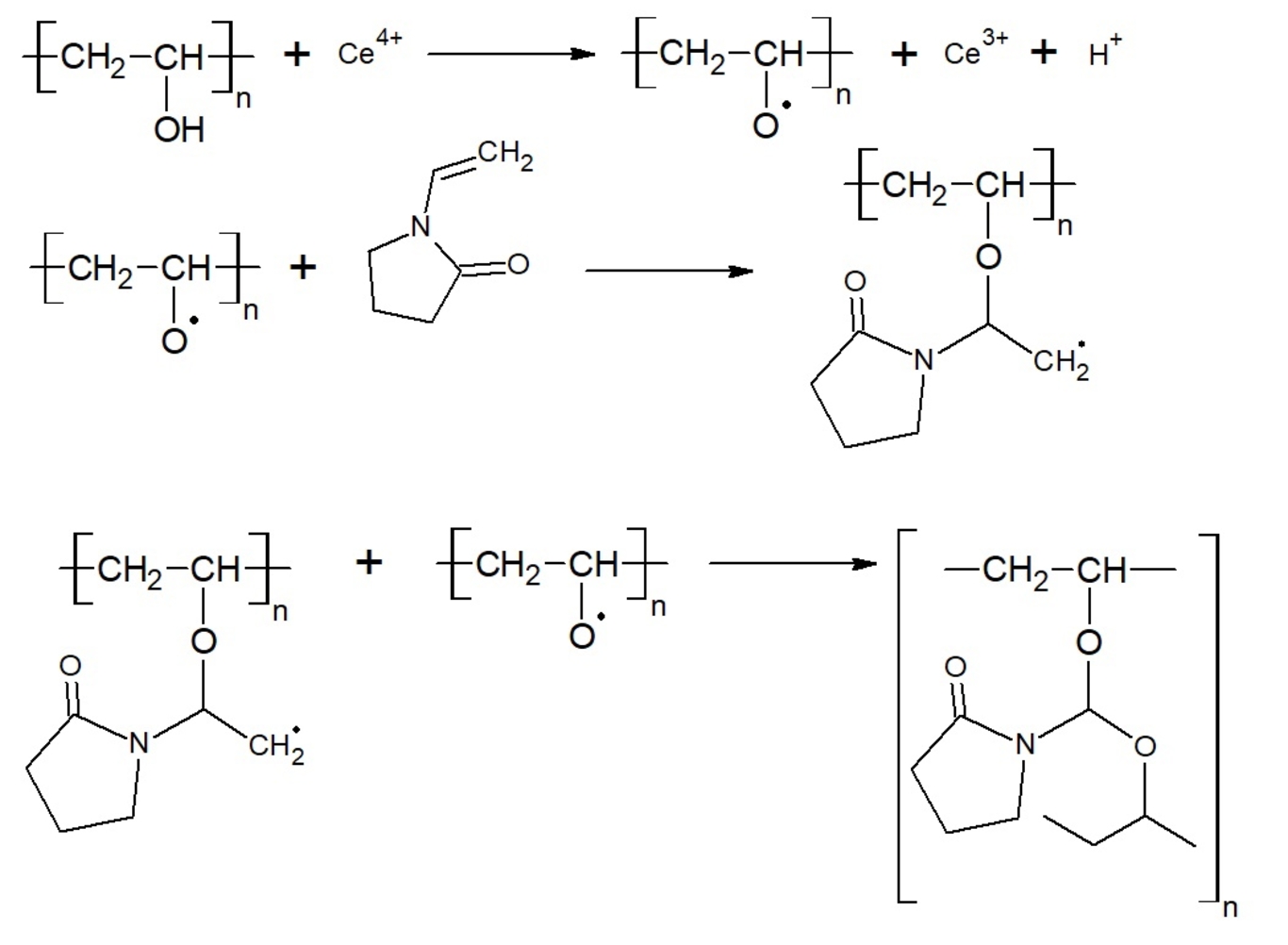

Supplement: Supplementary file 1 [file polymers-15-01296-s001.zip › polymers-2158334-supplementary/Supmat/Figure S1.jpg]

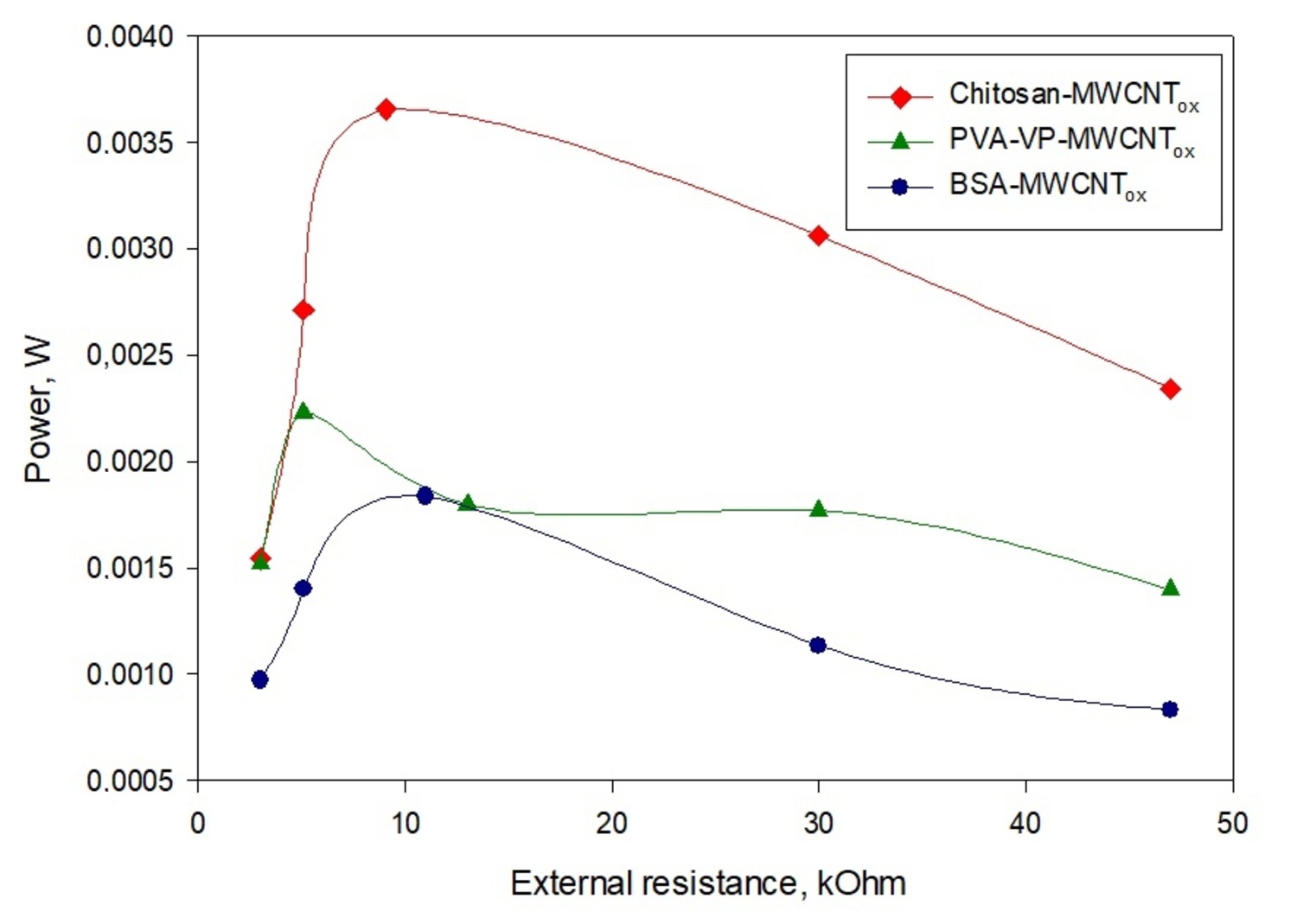

Supplement: Supplementary file 1 [file polymers-15-01296-s001.zip › polymers-2158334-supplementary/Supmat/Figure S2.jpg]

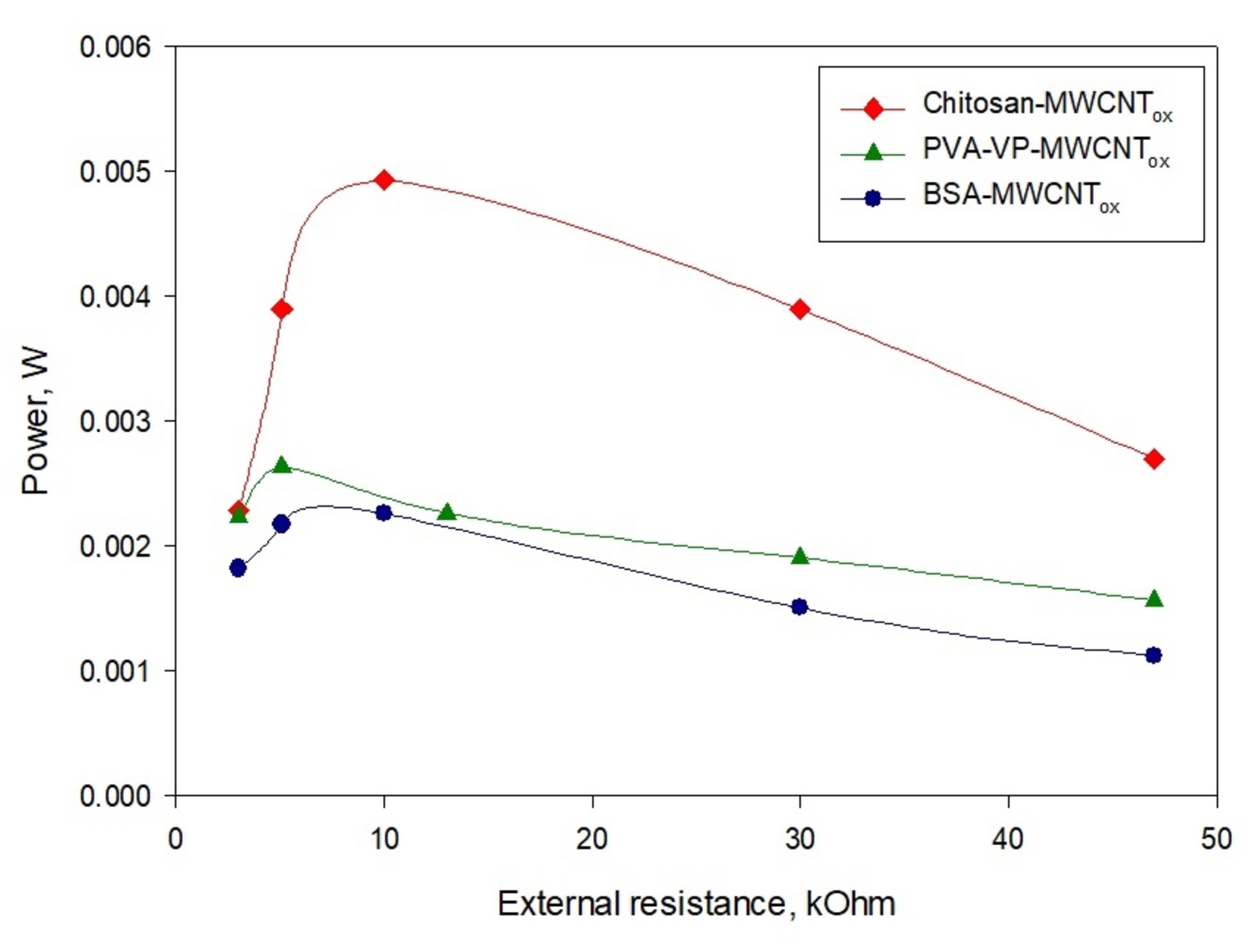

Supplement: Supplementary file 1 [file polymers-15-01296-s001.zip › polymers-2158334-supplementary/Supmat/Figure S3.jpg]
